# Global scaling of urban air quality

**DOI:** 10.1371/journal.pone.0333902

**Published:** 2025-10-13

**Authors:** Michael Wurm, Thilo Erbertseder, Matthias Weigand, Hannes Taubenböck

**Affiliations:** 1 Earth Observation Center (EOC), German Aerospace Center (DLR), Oberpfaffenhofen, Germany; 2 Department of Global Urbanization and Remote Sensing, Institute for Geography and Geology, Julius-Maximilians-Universität Würzburg, Würzburg, Germany; Lanzhou Jiaotong University, CHINA

## Abstract

Air pollution is a severe threat to urban residents worldwide. Growing cities and an increasing number of urban dwellers make urban air quality an important issue for the majority of the world’s population. While from economic and ecologic perspectives, extensive literature suggests that higher concentration of people in cities make urban areas more resource-efficient and environmentally sustainable, there is still unresolved ambiguity in the evaluation whether the same applies to urban air quality. Related case studies come to different findings, assuming larger cities to be either cleaner or more polluted than smaller cities in terms of air pollution; however, usually they consider only single countries which hinders generalizable answers to this question. Reasons for the variety of findings can be identified in the underlying data for air quality and in the varying spatial delineation of urban boundaries. In this study, we present a global analysis of urban air quality for more than 10,000 cities using scaling laws. Scaling laws define a relationship based upon a power law between the population size of a city and a certain characteristic of the city, e.g., in this study its level of air pollution. We rely on a satellite-derived globally homogenous data set for nitrogen dioxide (**NO**_**2**_) which is one of the major air pollutants and a proxy for air quality. Results reveal that globally, **NO**_**2**_ levels for cities scale linearly; however, certain regions and countries show strong superlinear and sublinear scaling behavior, indicating a strong regional dependency. We argue that one reason for this may lie in national policies for cleaner air in cities. Besides a homogenous data set for air quality, especially a harmonized definition for urban boundaries worldwide has proven to be of high importance for globally consistent and comparable results.

## 1. Introduction

Air quality is linked to human activities. Ambient air pollution poses, with an estimated 4.2 million deaths per year worldwide, a severe threat to the world’s population [1]. In particular, respiratory and cardiovascular diseases among others can be attributed to polluted air [2,3]. One of the major air pollutants is nitrogen dioxide (NO_2_), which mainly results from anthropogenic emissions produced by high-temperature combustion of fossil fuels (heating, power generation, industrial processes and engines in vehicles). Moreover, NO_2_ contributes to the formation of fine particulate matter 2.5 (PM_2.5_) and ozone, which are also harmful air pollutants [2–6].

Air pollution poses a threat especially to the population living in cities and with rising population size of a city, also the negative health impacts of air pollutants are increasing [7]. Because the number of global urban dwellers is estimated to reach up to 60% of the global population by 2030 [8], the effects of urban emissions on environmental and living conditions have been intensively debated [9–11]. Even though it might seem contradictory at first glance, there are ongoing scientific discussions whether larger cities are eventually environmentally ‘greener’ [12,13] due to higher densities and related reduced individual energy consumption and individual transport.

For the understanding of such complex processes, the size of cities is a key feature since many properties of cities have been found to be statistically related to the population size. In concrete terms, they are scaling as power law functions in relation to population size which helped to better understand cities as complex systems [14] and how infrastructure or social interactions can be related to the city size. In related studies, increasing returns to scale were observed for wealth and innovation as citizens in larger cities earn higher wages or more patents are being filed in larger cities [15]. At the same time, it was observed that urban infrastructure is subject to economies of scale [16] as, e.g., shorter road lengths or fewer gas stations are needed per capita in larger cities compared to smaller cities. With a growing body of literature dedicated to the scaling behavior of various urban parameters [16–18], also the scaling behavior of urban emissions with respect to city size has attracted increased attention to examine whether the air in larger cities is cleaner or more polluted than in smaller cities [19–25].

Unfortunately, however, the findings of these studies come to contradictory results. They describe larger cities as either dirtier in terms of emissions [24], greener or healthier [20,21,23], or they observe no particular effects with respect to population size [19]. The main sources for these confusions are found a) either in the data used for quantifying urban air quality, but more likely b) in the definition of the city itself [26], besides methodological ambiguities such as c) the used regression method [27].

Regarding a), major differences were observed in connection with the data sources used. Commonly, data from national emission inventories have been deployed [19,23,24], but these data can be problematic due to national biases resulting from bottom-up estimations which are subject to varying methodologies and data sources. Other studies are related to literature-based GHG emissions [25], or satellite-derived concentrations [21]. Another study estimated air pollution by distance traveled [20], but this has been found a critical proxy, as it may fall victim to underestimation of emissions due to congestion [22].

With regard to the second aspect of urban boundary definition b), satellite-based data of NO_2_ levels primarily comprise anthropogenic NO_2_ over urban areas, but also background NO_2_ in urban hinterlands [28]. In urban boundaries which comprise large but only sparsely populated areas, background NO_2_ can cumulate to significant amounts and may lead to biased scaling results. Therefore, the spatial delineation of urban areas is critical for the interpretation of results. A similar effect has also been reported for CO_2_ in US cities at different aggregation levels where the use of metropolitan statistical areas led to an overestimation of emissions, especially for smaller cities. This resulted in smaller allometric exponents than for urban areas which are delineated based on gridded population data [24,26].

Considering the lack of comparability of related studies due to the varying study designs and data, we address these issues in particular and present a global, systematic analysis of urban air quality scaling for more than 10,000 cities worldwide. The study design follows an explorative approach, in which we first test various data-related effects on urban air quality scaling (section 3) and then use these findings to analyze the global behavior of urban air quality scaling (section 4).

In particular, we focus in our study on the following objectives:

a) compare various *NO*_*2*_ *data sources* and their effects on urban scaling: we deploy global observations of NO_2_ tropospheric vertical column densities from three different earth observation satellites to account for varying spatial granularity of data. The sensors GOME-2B, OMI, and TROPOMI enable to yield gridded NO_2_ data sets at Level 3. In addition, we account for temporal effects such as comparing single-year data and composites of various years. Further, we compare the impact on the scaling behavior with the globally available bottom-up NO_x_-emission inventory EDGAR 5.0.
b) account for different *spatial definitions of cities* and their effects on scaling of urban air quality by comparing functional and morphological urban extents based on population density.

The outcome of this analysis results in a conceptual foundation for a global comparative study on urban air quality scaling by means of the proxy NO_2_. With it, we circumnavigate some of the most common fallacies in urban scaling by using a homogeneous data base for NO_2_-data and a harmonized definition of cities. In this way, we can:

c) analyze *global behavior on urban air quality scaling* with respect to geographic location.

In the remainder of the article, we begin by presenting the background on urban scaling and the used data in section 2. The impacts and the relevance of the data source and the city boundary definition is assessed in section 3 and the findings are applied within a global analysis in section 4. The results are discussed in section 5 and finally, section 6 concludes the paper.

## 2. Data and methods

### 2.1 Scaling laws for cities

Inspired by the observations of the allometric behavior between body mass and the metabolic rate of animals [29], also cities can be understood as metabolisms. In what has later become Kleiber’s law, he states that an organism’s metabolic rate scales to the ¾ of its mass meaning that larger animals are more energy-efficient. Later on, West [14] showed that this is related to the distribution networks like blood vessels that optimize energy delivery in organisms. He further described that also cities emerge from social and infrastructural networks such as people and roads and that they show similarities to metabolic systems. They have been found to follow allometric behaviors for many urban properties [16,18] following a ubiquitous urban scaling law:


$$Y\left(t\right)=Y_{o}\left(t\right){N(t)}^{\beta }$$ (1)

where Y(t) represents the urban property of interest (e.g., social activity or infrastructure) at time t. $Y_{o}\;$ is a normalization constant and the exponent β of this power law determines how strongly an urban property increases or decreases with city size N(t). If β=1, the urban property scales linearly with increasing population size, e.g., individual human needs such as total employment or total housing of a city. If β<1, we observe sublinear scaling, or economy of scale when less resources or infrastructure is needed per capita with increasing population size, e.g., number of gas stations, or road lengths. And, if β>1, we find a superlinear scaling relationship or increased returns with scale for social interactions, e.g., higher wages or more patents for larger cities [16]. In our analysis we aim at analyzing a potential sublinear or superlinear scaling relationship between per-city NO_2_ and population size:


$$E=CS^{\beta }$$ (2)

where E stands for the average NO_2_ of a city, C is the normalization constant, S is the city size measured as total population. After logarithmic transformation, this power law equation can be transformed into a linear regression function:


$${log}_{10}(E)={log}_{10}\left(C\right)+\beta \;{log}_{10}(S)$$ (3)

where β is the slope and 𝐥𝐨𝐠(S) is the intercept.

Regarding the scaling law relation between city size and air pollution, it has been argued that emissions might be higher in larger and less compact cities due to higher average commuting lengths [30]. On the other hand, the same authors also argue that compact cities might be responsible for higher emissions, because higher prices, wages and land rents, incentivize firms and households to change place which generates higher levels of pollution.

### 2.2 Urban boundaries and urban scaling

In earlier studies, urban scaling research was very much dependent on the availability of data on urban properties. In the course of time, however, new data sources at much higher spatial granularity have emerged which allow to disengage urban scaling research from traditional administrative boundaries [31] towards functionally interconnected regions such as functional urban areas [32], or morphological urban areas [33]. Today, many countries in the world use various spatial definitions, e.g., municipalities or metropolitan statistical areas (MSAs) in the US. These are based on counties defined as core urban areas due to their high population densities, and surrounding counties having strong economic links. Other spatial units include for example the *unités urbaines* (UU) in France which are based on population size and the settlement structure. This ambiguity in defining the city becomes of particular interest when urban scaling is applied for comparing cities from various countries in the world: by changing the spatial reference of the urban boundary, also the scaling behavior of various urban properties can be significantly affected [34,35] or even inverted [24,26].

To focus on human-related NO_2_-levels and to reduce the background NO_2_ in the data which results from large urban boundaries (e.g., from administrative units), we deploy a definition of urban areas which is based on population density and relates to the concept of the *degree of urbanization* of an area [36,37]. DEGURBA classifies urban areas into three types: cities (densely populated areas), towns and suburbs (intermediate density areas), and rural areas (thinly populated areas). This approach of clustering settlement areas has the advantage of global transferability because it is based only on population data which is usually available for cities worldwide. The original concept of DEGURBA was based on European local administrative units (LAU2) but was then extended to gridded population data from satellite-based urban footprints and census data. This helped to create a consistent, institutionally widely agreed (e.g., by OECD, EU, FAO, UN-Habitat) global data base on urban centers. The urban centers are defined as areas of *“contiguous grid cells of 1 km² with a density of at least 1500 inhabitants per km² and a minimum population of 50,000”.* The concept of DEGURBA is applied at global scale for the generation of the global human settlement footprint urban center data base (GHSL-UCDB) which contains > 10,000 urban centers with a population of > 50,000 inhabitants around the world [38]. The underlying satellite-based mapping of human settlements (GHSL) produces built-up maps from satellite images to derive spatial information about the presence of human activities over time [39]. Due to its unambiguous spatial definition and the manifold geographical and socio-economic contextual variables contained in the data set, we found the GHSL-UCDB an ideal data base for analysis of urban air pollution. For the subsequent analyses, only urban centers who passed the visual quality control procedure were employed, as described in [38]. These are automatically identified urban centers which were visually validated using a very high resolution map and a high-density settlement was found to be present. Further, urban centers from the Region of Melanesia were excluded because the population numbers in the UCDB data set were too high as they were from the districts and not from the cities themselves. In this way, we used 10,286 urban centers from the original 13,135 for the analysis.

### 2.3 Global estimates of nitrogen dioxide

In this study, we employ various sources on gridded NO_x_ data at different spatial granularities. In detail, we examine three sources of global observations of tropospheric NO_2_ column densities from space-borne sensors (TROPOMI, OMI, GOME-2) and gridded emission inventory data (EDGAR 5.0).

Satellite-based observations of nitrogen dioxide are established, globally consistent and objective data sources for examining spatial aspects of urban air quality [9,40–43] and analyzing trends of urban air quality over long time periods [44–48]. Since tropospheric NO_2_ predominantly results from anthropogenic combustion processes with small episodical contributions from lighting [49] and soil emissions [50] and it is chemically short-lived and therefore spatially bound to its sources, it constitutes a suitable proxy for urban air quality. Satellite data are represented as tropospheric vertical column densities [mol/m²] and were converted to the averaged total mass of NO_2_ per city over all available satellite orbits using the molar mass of NO_2_ (46 g) and each city’s area in Mollweide projection. Tropospheric NO_2_ observations were processed using HARP (Harmonization toolset for scientific Earth observation data; version 1.15; https://atmospherictoolbox.org/harp) and the ‘raster’ package in R [51] (version 3.6–30; https://r-packages.io/packages/raster). The tropospheric columns are often interpreted as proxies for ambient NO_2_ concentrations or air quality in general [11]. This relationship was substantiated, e.g., by [41]. Emission inventories represent the total NO_2_ emitted per grid cell, i.e., area unit per year. Consistently the emission data was converted to total NO_2_ per city per year. We do not, however, directly compare emission data with tropospheric columns since we aim at analyzing the effects on the allometric scaling behavior of each data set.

#### TROPOMI.

The TROPOspheric Monitoring Instrument (TROPOMI) is mounted aboard Sentinel-5 Precursor (2017 to present) and allows for a daily global coverage at the highest available spatial resolution at an equator crossing time at 13:30 hrs [52]. Observations of tropospheric NO_2_ vertical column densities have a spatial resolution of 3.5 km x 7.5 km (across x along track) and of 3.5 km × 5.5 km since 2019 [53]. Each orbit (Level 2 products of version 1.2) was oversampled onto a grid of 0.025° × 0.025° longitude-latitude resolution to enable a consistent averaging and data comparison [9].

#### OMI.

The Ozone Monitoring Instrument (OMI) on board of NASA’s AURA satellite is a nadir-viewing spectrometer on a polar sun-synchronous orbit with a local equator crossing time at 13:45 hrs [54]. Global daily data on tropospheric NO_2_ vertical column densities are available since 2004 at a spatial resolution of 13 km x 24 km at nadir. We apply tropospheric NO_2_ vertical column densities (QA4ECV version 1.1) at a spatial sampling of 0.125° × 0.125° (Level 3) [55].

#### GOME-2B.

The Global Ozone Monitoring Experiment-2 (GOME-2) aboard MetOp-B (2012 to present) is a nadir-scanning spectrometer with an equator crossing time at around 9:30 hrs local time [56]. Ground pixel size is 80 km × 40 km [56] and tropospheric NO_2_ vertical column densities are retrieved operationally following Valks et al. [57]. All available orbits as Level 2 offline products of version 4.8 were sampled onto a global grid of 0.25° × 0.25° geographical resolution.

#### EDGAR v5.0.

To further test our hypothesis, we also consult gridded emission inventory data. The Emissions Database for Global Atmospheric Research (https://edgar.jrc.ec.europa.eu/index.php/dataset_ap50) provides yearly and sector-specific gridmaps for nitrogen oxide (NO_x_) at a spatial sampling of 0.1° × 0.1° in the period 1970–2015 [58,59].

## 3. The role of input data characteristics on scaling estimates

**Fig 1:**
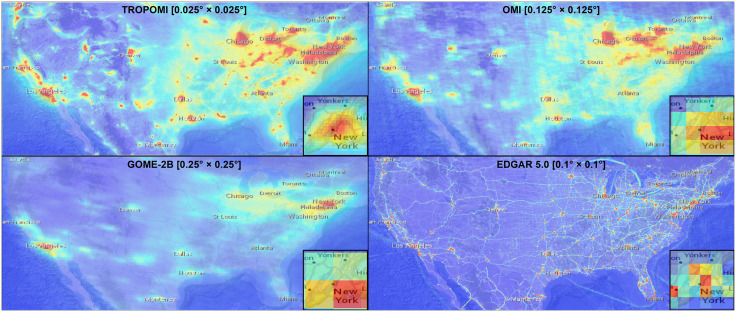
Visual representations of NO_2_ data for the year 2019 and for the entire continental U.S. (inset: New York City). Panels show data from four sources TROPOMI, OMI, GOME-2 and EDGAR, highlighting their differences in spatial resolution. Fine-granulated data from TROPOMI reveals polluted areas and reveals spatial decreasing gradients of air pollution from city centers towards the outskirts. OMI, with only 1/25 of TROPOMI’s spatial resolution still identifies the centers of air pollution while the number of individual data points for each city decreases noticeable. For GOME-2, however, the 1/100 loss of resolution makes patterns of air pollution still visible across the US, and large urban areas but finer spatial determination becomes impossible. The spatial structure of NO_x_ emissions from EDGAR is clearly related to human infrastructure and urban areas which are used for spatial disaggregation of emission inventory data. Base map and data from OpenStreetMap and OpenStreetMap Foundation.

Nitrogen dioxide pollution for each city under investigation is calculated from *gridded NO*_*2*_ *data* for each *urban entity.* The spatial granularity of the global NO_2_ data varies between 0.025° × 0.025° (~2.8 km) for TROPOMI, 0.125° × 0.125° (~14 km) for OMI and 0.25° × 0.25° (~28 km) for GOME-2. Naturally, due to the varying granularity the number of data observations per city under investigation also varies significantly (Fig 1). This can lead to effects of ecological fallacy or the modifiable areal unit problem MAUP [60], especially for very small urban areas. Considering the spatial variations of these data sets, we aim at identifying which of the available data sets for ambient urban air quality and also which urban boundary definition is best suited for global analyses. Therefore, we first perform the following analyses for a spatial subset of 321 urban centers within the continental U.S. to test various parameters at national scale.

### 3.1 Spatial granularity and data source

First, we compare the various NO_2_ data sets with respect to their original spatial granularity for the year 2019. This is the only year for which all satellite data under investigation is available prior to disruptions caused by the COVID-19 pandemic [61]. Emission data from EDGAR was selected from 2015, as it was the most recent available year at the time this study was conducted. We acknowledge, however, the temporal mismatch between EDGAR and the satellite data. We first analyze each data source with regard to their ability to capture nitrogen oxides under consideration of their individual granularity. To overcome spatial bias in the data sets (Fig 1) and to preserve original values, all raster grids are resampled to a very high spatial resolution of 0.0125° × 0.0125°. We do so, as this value is a fraction of the resolution of TROPOMI and GOME-2B and corresponds to the resolution of OMI. Resampling is performed using the nearest neighbor algorithm, because it is computationally very efficient and preserves the original pixel values. This is of advantage when various data sets are compared with each other. The resulting pixels are then superimposed with the urban boundaries to retrieve the average NO_2_ tropospheric column in mass (kg) for each city.

Comparing the retrieved values reveals that both TROPOMI and OMI follow a very similar value distribution despite their differing original resolution of a factor of 1:25 (Table 1). We conclude that despite the varying spatial granularity of the data, OMI is capable of capturing city-wide NO_2_ pollution almost as precisely as the much higher resolved TROPOMI data. For lower resolved GOME-2B (factor 1:4 compared to OMI and factor 1:100 compared to TROPOMI), however, we observe significantly lower NO_2_ values, which are only about 50−70% of those from OMI and TROPOMI. The coarse native resolution of GOME-2B and subsequent downscaling from 0.25° to 0.125° explain only part of the discrepancy, as degrading of high-resolution TROPOMI and OMI data to 0.25° changed city-level NO₂ values only marginally (Table 1). Moreover, the different orbits and overpass times between GOME-2, OMI and TROPOMI, respectively, lead to diurnal variation and varying vertical column densities.

**Table 1:** Descriptive statistics of satellite-retrieved NO_2_ tropospheric mass per city area [in kg] for 321 continental U.S. metropolitan areas in 2019 in original data resolution and coarsened to the lowest available resolution (0.25°).

	Min.	1^st^ Qu.	Median	Mean	3^rd^ Qu.	Max.
**TROPOMI [0.025°]**	25.9	85.4	153.9	743.1	332.1	32,308.1
**TROPOMI [0.25°]**	25.0	77.7	140.8	680.4	306.7	28,796.2
**OMI [0.125°]**	26.9	83.9	152.8	767.5	384.0	35,825.4
**OMI [0.25°]**	28.0	85.3	151.6	750.8	366.1	34,291.8
**GOME-2B [0.25°]**	5.1	43.9	84.8	437.2	215.0	23,043.5

**Fig 2:**
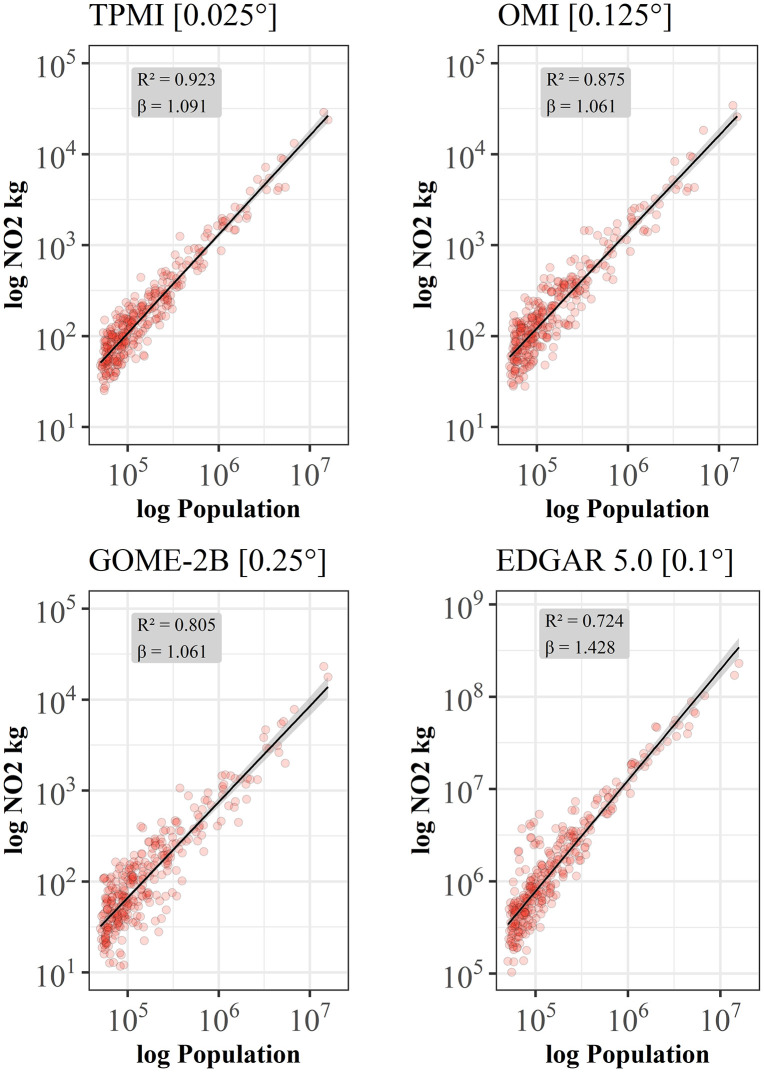
Scaling of satellite-based NO_2_-air quality and inventory-based NO_2_-emissions for 321 continental U.S. cities. Data for urban areas were obtained from satellite data for the year 2019, and emissions from EDGAR 5.0 from 2015, and urban boundaries from GHSL-UCDB (red dots; cf. section 3.2). Black lines show the best fit to a scaling relation $𝐘\left(t\right)=Y_{o}\left(t\right){N(t)}^{\beta }$, with β = 1.091 ± 0.035 [95% confidence interval (CI), Adj. R² = 0.923] for TROPOMI, β = 1.061 ± 0.045 [95% confidence interval (CI), Adj. R² = 0.875] for OMI, and β = 1.061 ± 0.058 [95% confidence interval (CI), Adj. R² = 0.805] for GOME-2B, and β = 1.200 ± 0.051 [95% confidence interval (CI), Adj. R² = 0.874] for EDGAR.

For all 321 continental U.S. cities in the data set, the scaling relationship between NO_2_ and population size is analyzed using scaling laws (see section 2.1). The resulting scaling estimates are presented as log-log plots of city-level NO_2_ mass compared to the population size for the three satellite sensors in Fig 2. We find superlinear scaling for all sensors with small effects of input data granularity: For TROPOMI the highest scaling exponent *β = 1.091* (*R² = 0.923*) is reported, while for both other sensors the exponent is with *β = 1.061* (OMI: *R² = 0.875;* GOME-2B: *R² = 0.805*) marginally lower. Scaling relations are reported stable with regard to decreased data resolution since despite the significantly reduced granularity, both, the coefficient of determination and the scaling exponent are only negligibly lower (Table 2).

**Table 2:** Summary of the estimated scaling exponents for the various spatial granularities of NO_2_ data.

	*β*	R²	CI
**TROPOMI [0.025°]**	1.091	0.923	± 0.035
**TROPOMI [0.25°]**	1.086	0.917	± 0.036
**OMI [0.125°]**	1.061	0.875	± 0.045
**OMI [0.25°]**	1.058	0.876	± 0.044
**GOME-2B [0.25°]**	1.061	0.805	± 0.058
**EDGAR**	1.200	0.874	± 0.051

To account for data source-related scaling effects between satellite-based observations and inventory-based NO_2_ as used in some studies [19,24,26], we also analyze the scaling relation from EDGAR 5.0 in its original spatial resolution of 0.1° × 0.1° (Fig 2). Reported values from EDGAR are in total NO_x_ per year and thus are generally higher than values for NO_2_ averages [45]. Besides this observed higher level in general, the scaling relation with population reports also a superlinear relation but with a significantly higher scaling exponent of *β = 1.200* compared to satellite-based scaling estimates. Hence, according to reported emission inventory data, the air quality of larger cities in the U.S. is much more affected than in smaller cities compared to satellite-based measurements (Table 2).

Effects of input data granularity can be observed only regarding city-wide NO_2_ levels, but the scaling exponents are only affected to a minor degree (Fig 2) between TROPOMI and OMI. Therefore, and because of its global coverage and the long-term data availability over many years, OMI data represent a valuable and effective data source for global analyses of urban air quality scaling.

### 3.2 Urban boundary definition

**Fig 3:**
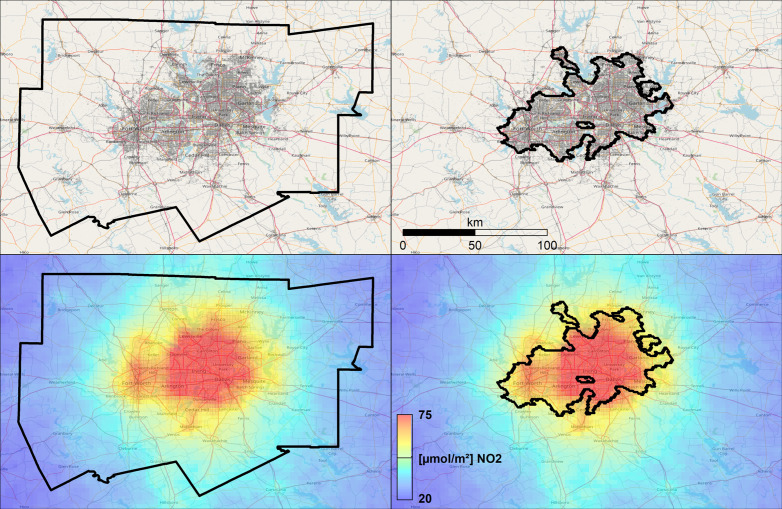
Effects of urban boundary definition on inferring satellite-based burdens of NO_2_. Top row displays on the left the boundary of the Metropolitan Statistical Area (MSA) for Dallas-Fort Worth-Arlington, TX (area: 24,100 km²; population: 7.04 million), and on the right the morphologic urban boundary from GHSL-UCDB (area: 3,700 km²; population: 5.16 million) superimposed over © Open Street Map data and impervious surfaces from the World Settlement Footprint [62]. Bottom row displays the urban boundaries superimposed over NO_2_ tropospheric columns from TROPOMI instrument at full spatial sampling of 0.025° × 0.025°. The different spatial extents of urban boundaries result in significantly differing integrated NO_2_ burdens: a total of 39.2 t NO_2_ for the MSA and 9.2 t NO_2_ for the morphologic boundary, respectively. In combination with the respective population, this results in 5.6 kg NO_2_ per capita for MSA and 1.8 kg NO_2_ per capita for UCDB. Base map and data from OpenStreetMap and OpenStreetMap Foundation.

A strong impact on the calculation of the scaling exponent is induced by the spatial definition and the size of urban boundaries. Oliveira et al. [24] report significantly varying scaling results for ambient air quality using varying urban definitions. The variation of political and administrative boundaries in the context of urban air quality is depicted in Fig 3, visualizing the spatial differences compared to morphological boundaries based on population density and impervious surfaces from UCDB [38]. It also reveals the significant impact of background NO_2_ on the total sum of NO_2_ for the urban area under observation.

**Fig 4:**
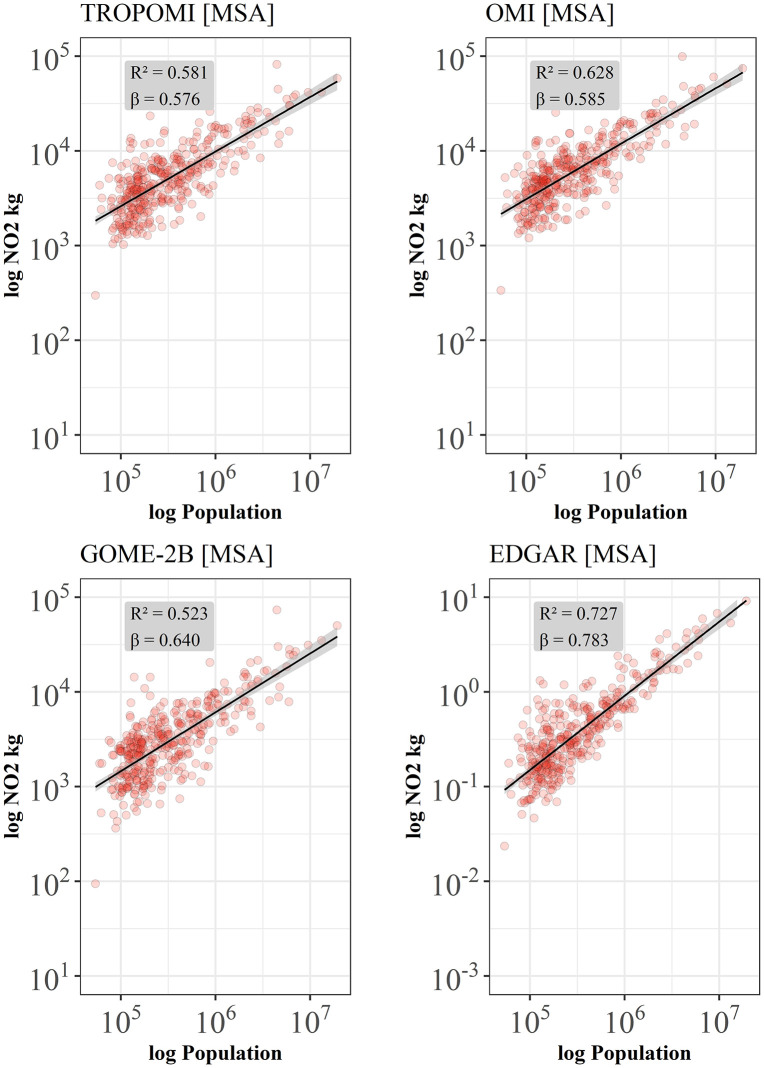
Sublinear scaling of satellite-based NO_2_-amounts and inventory-based NO_x_ emissions for continental U.S. Metropolitan Statistical Areas (MSA). Data for 377 MSAs (red dots) were obtained from satellite data for the year 2019, and emissions from EDGAR 5.0 from 2015. Black lines show the best fit to a scaling relation $𝐘\left(𝐭\right)={𝐘}_{𝐨}\left(𝐭\right){𝐍(𝐭)}^{\beta }$, with β = 0.576 ± 0.050 [95% confidence interval (CI), Adj. R² = 0.581] for TROPOMI, β = 0.585 ± 0.046 [95% confidence interval (CI), Adj. R² = 0.628] for OMI, and β = 0.640 ± 0.063 [95% confidence interval (CI), Adj. R² = 0.523] for GOME-2B, and β = 0.783 ± 0.049 [95% confidence interval (CI), Adj. R² = 0.727] for EDGAR. All estimated exponents are sublinear, and estimates are statistically robust.

Here, we compare the impact of urban boundary definitions on the scaling estimates between administrative boundaries from Metropolitan Statistical Areas (MSA) and morphological boundaries based on population density and impervious surfaces from the urban center data base (GHSL-UCDB): reported scaling exponents *β* and coefficients of determination *R²* are significantly lower for all data sets when the scaling is performed using urban boundaries from MSAs (Fig 4) compared to morphological boundaries from GHSL-UCDB (Fig 2). All results for MSA report strong sublinear scaling exponents between *β* = 0.576 (TROPOMI), *β* = 0.585 (OMI), *β* = 0.640 (GOME-2B), and *β* = 0.783 (EDGAR) which would indicate a strong NO_2_ saving for larger cities.

Combustion processes are relevant for the production of nitrogen dioxides, and these are local phenomena (Fig 3). Thus, the selection of a suitable spatial unit which captures the spatial extent of populated areas and thus the sources of air pollution is important. This is because too large administrative areas incorporate more background NO_2_ and thus may impact the scaling exponents from superlinear to sublinear which leads to divergent conclusions. Reported very high coefficients of determination *R²* for scaling of NO_2_ using morphological boundaries (Table 2) reinforce this appraisal.

### 3.3 Temporal effects: Decreasing NO_2_, increasing population, and meteorological effects

For global analyses of ambient air quality based on satellite measurements, variations of observed NO_2_ levels over time are to be considered. Causes for these variations are mostly due to two effects: 1) short-term meteorological effects [63] or exogenic effects such as global shutdowns of human activity and mobility [61,64], and 2) overlying, long-term trends in NO_2_ reduction due to, e.g., increased environmental awareness.

**Fig 5:**
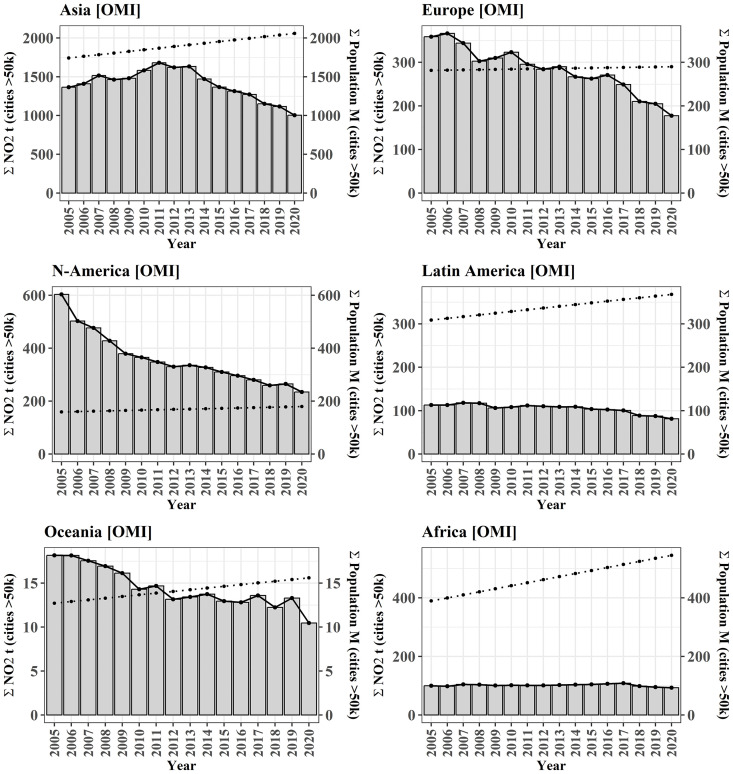
Temporal evolution for NO_2_ and population trends for each continent for the cities of this study. Data shows total NO_2_ burden from OMI in t for 2005-2020 (bars and black line) and total population (dotted trend line) of all cities with a minimum population size of 50,000 inhabitants. Population data was predicted based on a linear model between known population counts for 2000 and 2015, as reported in the UCDB. These trends show a decoupling between increasing populations and decreasing NO_2_, especially in higher income regions.

Over the last decades, varying trends of NO_2_ in cities are observed [44,47,65]. There is a general decrease for all continents in recent years with varying amplitude, and shape, as the bars in Fig 5 indicate. While Europe (−50.5%), Northern America (−61.2%), and Oceania (−42%) are measured with almost linear decreases over the entire monitoring period, NO_2_ in Africa has been almost stable for 15 years (−6.6%). For Asia (−26.4%), and Latin America (−27.9%), the decreasing trend has been established with some delay only after 2013 and 2014, respectively. This can mostly be related to the delayed implementation of environmental regulations reducing emissions from motor vehicles and industry. In terms of data analysis, proposed strategies for mitigating such effects of interannual variability in NO_2_ levels, include averaging data from multiple years [9,47,66–68]. The temporal scale over which data is averaged, is important to avoid biased results in the scaling analysis. This is also because in the same time period, the other parameter for scaling analysis, the population counts in the cities of each continents, evolved differently. For population almost no increase is reported for Europe (+3.0%), low to medium rates are reported for Northern America (+13.1%) and Latin America (+19.2%), Asia (+18.2%), Oceania (+23.1%), and highest increase can be observed for Africa (+40.0%) (dotted line in Fig 5). To address these issues together with additional impacts on the emissions from COVID-19 lockdowns, and to minimize short-term fluctuations, the years 2017–2019 are used for global analysis.

## 4. Global analysis of urban air quality scaling

Here, we apply the previously tested set-up to analyze how air quality in cities scales globally using a 3-year aggregate (2017–2019) of OMI satellite-based observations in combination with extrapolated population data for 2018, and morphological urban boundaries following a harmonized concept based on impervious areas and population density. We test previously formulated theories on variations in urban air quality scaling, e.g., for geographic regions, socioeconomic factors, the countries’ economic power, or climate [24,25,47,69].

**Fig 6:**
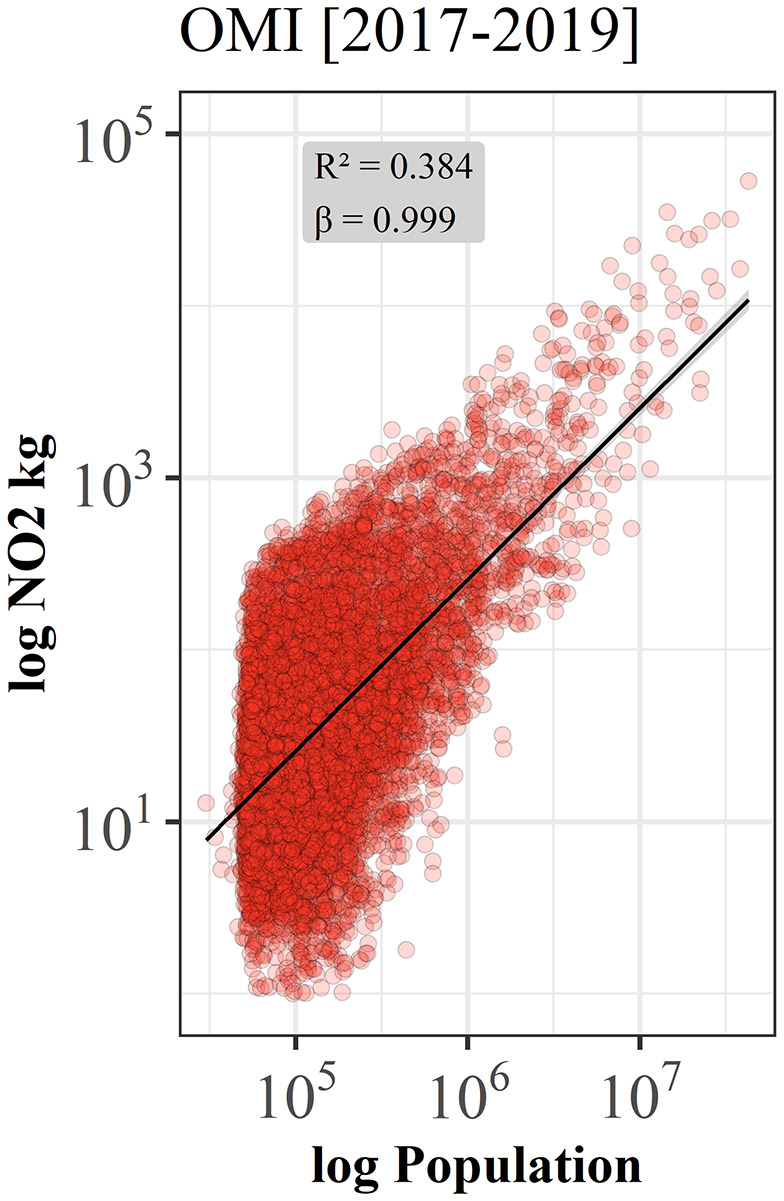
Scaling of urban air quality for 10,286 urban centers around the world. For the analysis, averaged NO_2_ levels from OMI for the period 2017-2019 were used and the best fit to a scaling relation (black line) $𝐘\left(𝐭\right)={𝐘}_{𝐨}\left(𝐭\right){𝐍(𝐭)}^{\beta }$ with β = 0.999 ± 0.025 [95% confidence interval (CI), Adj. R² = 0.384].

Without any regionalization, urban air quality scaling at global level reports a linear scaling relation (*β = 0.999*) which represents a good baseline for the following analyses of geographic sub-regions since no significant deviation from linearity in global scaling behavior was observed. The lower R² however, can be interpreted as an indication that cities in some regions in the world would scale differently. At a global scale, however, larger cities are neither more polluted nor cleaner than smaller cities (Fig 6).

### 4.1 Spatial entities: Are some regions cleaner than others?

**Fig 7:**
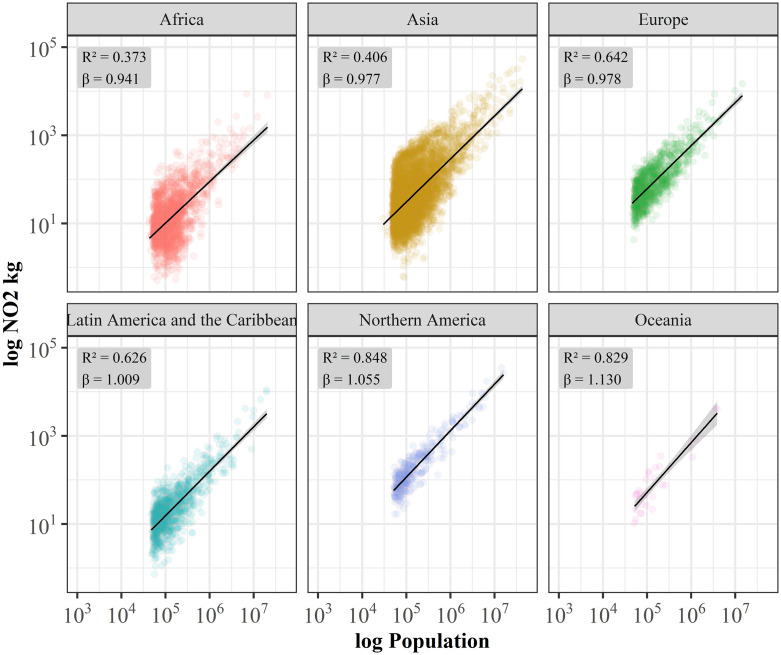
Urban scaling relation of NO_2_ grouped by continents. Data shows averaged NO_2_ levels from OMI satellite measurements for the period 2017-2019 for 10,286 cities in the world from the UCDB (colored dots) and the best fit to a scaling relation (black lines) $𝐘\left(𝐭\right)={𝐘}_{𝐨}\left(𝐭\right){𝐍(𝐭)}^{\beta }$ with β = 0.941 ± 0.054 [95% confidence interval (CI), Adj. R² = 0.373] for Africa (n = 2028), β = 0.977 ± 0.031 [95% confidence interval (CI), Adj. R² = 0.406] for Asia (n = 5760), β = 0.978 ± 0.045 [95% confidence interval (CI), Adj. R² = 0.642] for Europe (n = 1051), β = 1.009 ± 0.048 [95% confidence interval (CI), Adj. R² = 0.626] for **L.** America & Carib. (n = 1045), β = 1.055 ± 0.046 [95% confidence interval (CI), Adj. R² = 0.848] for Northern America (n = 372), β = 1.130 ± 0.175 [95% confidence interval (CI), Adj. R² = 0.829] for Oceania (n = 35).

In section 3.1 we found strong correlation (*R² = 0.857*) and weak superlinear scaling relation (*β = 1.061*) for the United States. Here, we extend this analysis to all continents (Fig 7). Results vary and report strong superlinear scaling behavior for Oceania (*β = 1.373*), and weak superlinear scaling for Northern America (*β = 1.055*) while Latin America (*β = 1.009*), Europe (*β = 0.978*), and Asia (*β = 0.977*) scale linearly, and Africa (*β = 0.941*) shows weak sublinear scaling, however, with lower coefficients of determination.

**Fig 8:**
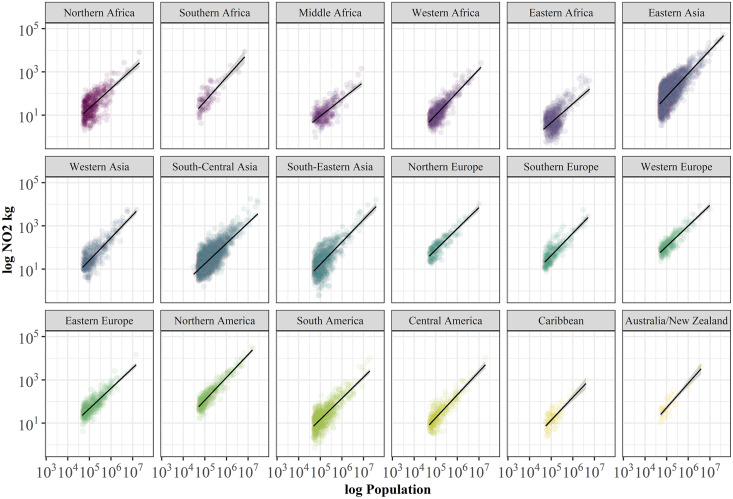
Scaling relation of levels of NO_2_ amounts grouped by Geographic Regions of the world. Data shows averaged NO_2_ levels from OMI satellite measurements for the period 2017-2019 for 10,286 cities in the world from the UCDB (colored dots) and the best fit to a scaling relation (black lines) $𝐘\left(t\right)=Y_{o}\left(t\right){N(t)}^{\beta }$. Scaling exponents and coefficients of determination are reported in Table 3. Geographic regions are defined according to [8].

From a naïve geographic perspective, using continents for grouping the cities seems to be a plausible approach. A deeper understanding of the internal differences in, e.g., varying development states and economic power or climatic conditions of the comprised countries, however, demands a more tailored geographic grouping. Since in our empirical study we are looking for characteristics of cities in different regions of the world in which air pollution is remarkably lower or higher in larger cities than in smaller cities, in the following analysis we break down the continents into Geographic Regions. The Geographic Regions are a concept used by the United Nations [8] and divide each of the six continents into geographic sub-regions. Related results reveal remarkably differing intra-continental scaling coefficients compared to the entire continents (Fig 8 and Table 3) and clearly underline the importance of spatial entities when drawing conclusions at the global or continental scale. While NO_2_ scales sublinearly for the entire African continent (*β = 0.941*) (Fig 7), intra-continental regions such as Southern Africa (*β = 1.118*) or Western Africa (*β = 1.055*) report superlinear scaling. At the same time, Middle Africa (*β = 0.788*) and Eastern Africa (*β = 0.871*) report lowest exponents. Similar differences are observed for Asia which scales sublinearly for the entire continent (*β = 0.977*), but at the level of Geographic Regions, only South-Central Asia (*β = 0.941*) report sublinear scaling while the remaining three regions scale superlinearly. In Europe (*β = 0.978*), only Southern Europe is bucking the general sublinear trend with a superlinear scaling behavior (*β = 1.032*). The almost linear scaling of Latin America (*β = 1.009*) represents a good example when superlinear Central America (*β = 1.068*) and Caribbean (*β = 1.055*) are averaged out by sublinear South America (*β = 0.979*). Oceania is only represented by Australia and New Zealand (*β = 1.113*). Micronesia, Polynesia and Melanesia are ignored due to poor data quality.

**Table 3:** Coefficients for scaling results by regions of the world.

Region	β	Adj. R²	CI	n
*Northern Africa*	0.905	0.424	0.098	461
*Southern Africa*	1.118	0.642	0.177	89
*Middle Africa*	0.788	0.510	0.094	271
*Western Africa*	1.055	0.687	0.055	662
*Eastern Africa*	0.871	0.319	0.109	545
*Eastern Asia*	1.068	0.531	0.044	2060
*Western Asia*	1.043	0.686	0.072	385
*South-Central Asia*	0.941	0.555	0.033	2581
*South-Eastern Asia*	1.037	0.490	0.078	734
*Northern Europe*	0.977	0.725	0.089	182
*Southern Europe*	1.032	0.696	0.090	230
*Western Europe*	0.949	0.764	0.070	227
*Eastern Europe*	0.937	0.713	0.059	412
*Northern America*	1.055	0.848	0.046	372
*South America*	0.979	0.593	0.060	720
*Central America*	1.068	0.715	0.085	253
*Caribbean*	1.055	0.665	0.177	72
*Australia/New Zealand*	1.130	0.829	0.175	35

**Fig 9:**
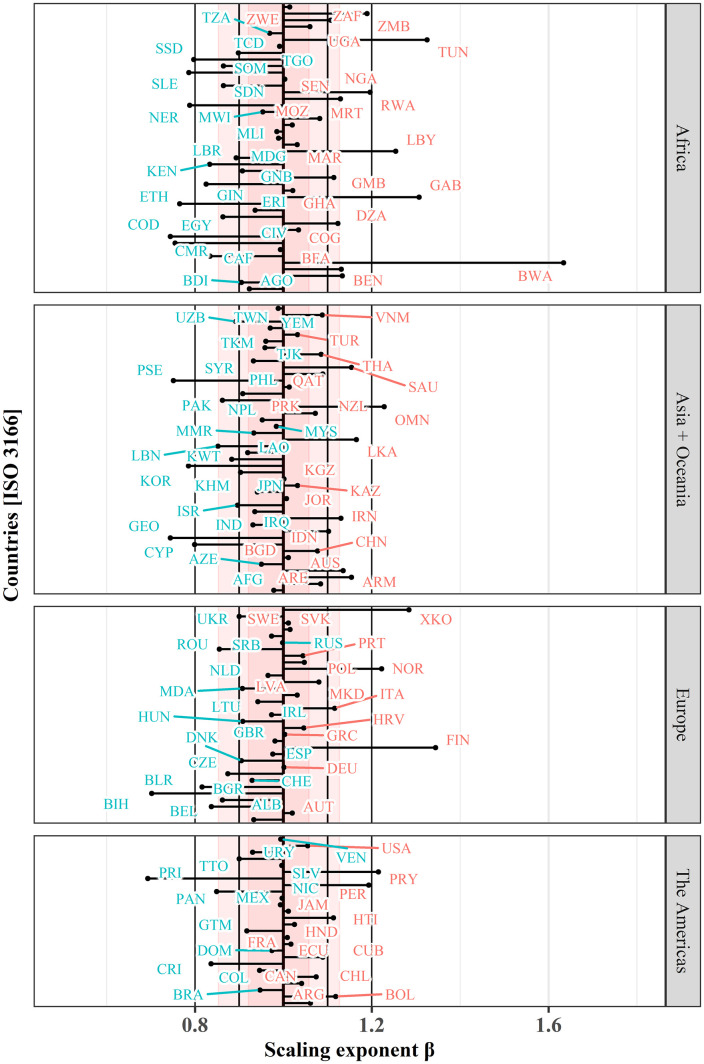
Comparison of scaling exponents for all countries, grouped by aggregated continents. Country names and detailed representation of scaling exponents, coefficient of determination and confidence interval are presented in detail in (S1 Table). Thick black line represents linearity (β = 1), red bands represent σ and 2 × σ.

Breaking the spatial entities further down for the scaling analysis, the finest-grained analysis of cities’ air quality scaling behavior can be performed at the spatial entity of the 155 individual countries with more than two cities with more than 50,000 inhabitants (Fig 9). Analysis at the individual country level allows for finding more causal factors such as policy implications of countries on the scaling behavior of cities.

Central tendency of the scaling analysis reports marginal sublinear scaling behavior for all countries with a median of *β*_*md*_* = 0.988*, a mean *β*_*mn*_* = 0.962*, first and third quantile are reported with *β*_*Q1*_* = 0.902* and *β*_*Q3*_* = 1.073*, respectively. The distribution shows that 86 countries (55.1%) report scaling coefficients between **0.9 < *β* < 1.1**. 50 countries (32.1%) are close to linearity **0.95 < *β* < 1.05**, and the majority of all countries (55%) report sublinear scaling *β < 1.*

The variations of scaling estimates for the countries contained in the four continents reveal that no geographical association between the scaling exponents and the continent can be observed, e.g., for all continents we find countries with very high but also with very low *β*. This observation is also supported by variations of *β* which range between *σ = 0.11* for Asia + Oceania/ The Americas, *σ = 0.13* for Europe, and *σ = 0.18* for Africa.

The highest reported exponents and thus the largest increase of the NO_2_ burden with increasing city size are measured for Botswana (*β = 1.634*), Tunisia (*β = 1.355*), Finland (*β = 1.344*), Gabon (*β = 1.307*), Kosovo (*β = 1.284*), Libya (*β = 1.254*), Oman (*β = 1.228*), and Norway (*β = 1.222*). Botswana appears to be a strong statistical outlier which can be explained by the very high NO_2_ burden per capita for the country’s capital Gaborone. The capital is with 0.497g higher than the per capita NO_2_ burden of 72% of all the cities in the world. Besides a disproportionately high concentration of industry and transport related emissions at the country’s economic center, the close geographical vicinity to the heavily polluted air of the Highveld plateau in South Africa [70] could provide additional explanation for this phenomenon. For most of the other countries with very high superlinear scaling behavior, the majority can be associated with the Global South; however, especially Finland and Norway appear unexpectedly although they are representatives of high-income countries with well-developed ecological consciousness and political agendas to reduce carbon emissions. It has been argued that high, and even rising NO_2_ levels in the Nordic countries can be partly explained by the increasing usage of diesel cars due to newly introduced CO_2_ emission-based tax [71,72], which underpins the importance to consider political decisions in the interpretation of scaling behavior of urban air quality. Moreover, this effect could be induced as well due to an observational bias due to the countries’ northern geolocation and the limited number of observations in winter.

The ‘greenest countries’ with lowest scaling coefficients are Puerto Rico (*β = 0.693*), Bosnia and Herzegovina (*β = 0.702*), Georgia (*β = 0.744*), Democratic Republic of the Congo (*β = 0.744*), Palestine (*β = 0.751*), Cameroon (*β = 0.755*), Ethiopia (*β = 0.765*), and Western Sahara (*β = 0.784*). The majority are countries located in Africa or in the Global South in general. However, at the bottom side of scaling exponents, we also find South Korea, a highly industrialized and wealthy country with the world’s tenth-largest nominal GDP, but also an increased usage of diesel vehicles [73]. NO_x_ emissions, however, have been effectively decreased due to pollution control policies targeting vehicular emissions in the Seoul Metropolitan Area [74].

Synoptically, in the light of these observations of varying scaling behavior for various aggregates of geographical entities, we find no unambiguous association of scaling exponents for all countries of a continent or geographical region. At the level of individual countries, we find both, highly industrialized countries and countries of the Global South on both ends of the range of exponents, which negates any direct, plausible relation between air quality scaling and geographic location but rather underpins the significant role of national ecological policies to provide a healthy environment for its citizens.

### 4.2 Socio-economic impacts: Do richer countries exhibit cleaner urban air due to more efficient scaling?

**Fig 10:**
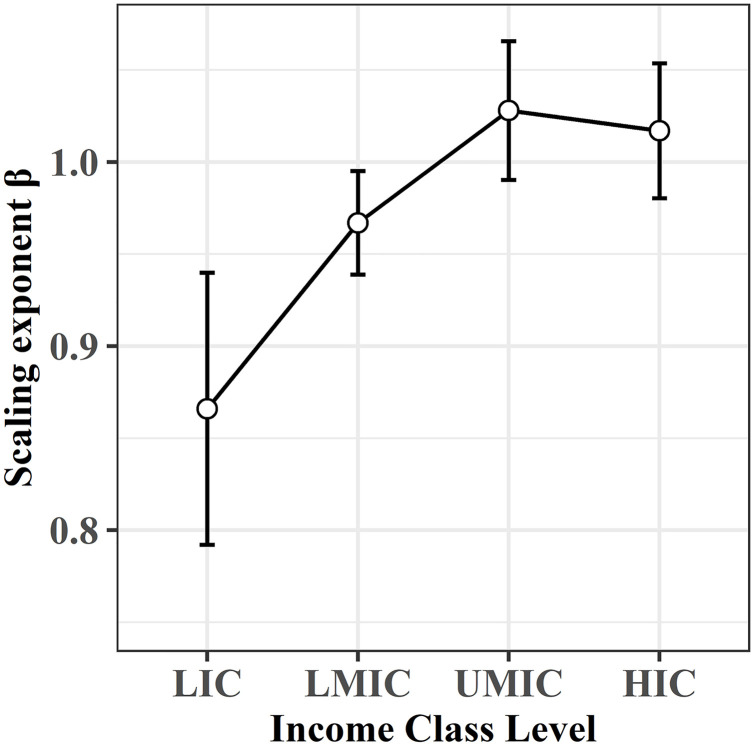
Environmental Kuznets Curve for scaling exponents and Income Class Levels including confidence intervals. Exponents rise between Low-Income Class and Upper Middle-Income Class, and then decrease for countries of High-Income Class.

In search of a causal relationship between environmental quality and economic development, the Environmental Kuznets Curve (EKC) has provided empirical explanations why some indicators of environmental behavior get first better and then worse with rising economic growth [75]. The EKC describes an inverted u-shaped relationship between environmental degradation and wealth. It indicates that for lower income countries increased pollution is observed and beyond a certain level of wealth, the trend reverses because of environmental improvement [76]. The application and validity of the inverted u-shaped curve has since then been controversially discussed [76], but its applicability has been positively evaluated for some ecological indicators including air quality [69,77]. Some authors found empirical evidence of its applicability for selected geographical regions also in the context of urban air quality scaling [24,25]. Based on these indications, we group the countries based on their income class level [8] for the analysis of scaling behavior in relation to the countries’ economic power (Fig 10): high-income class (HIC), upper middle-income class (UMIC), lower middle-income class (LMIC), and low-income class (LIC). Our findings exhibit an EKC-like pattern, characterized by scaling exponents that increase with income up to upper-middle-income levels, subsequently decreasing for high-income countries (Fig 10).

### 4.3 Influence of population data

**Fig 11:**
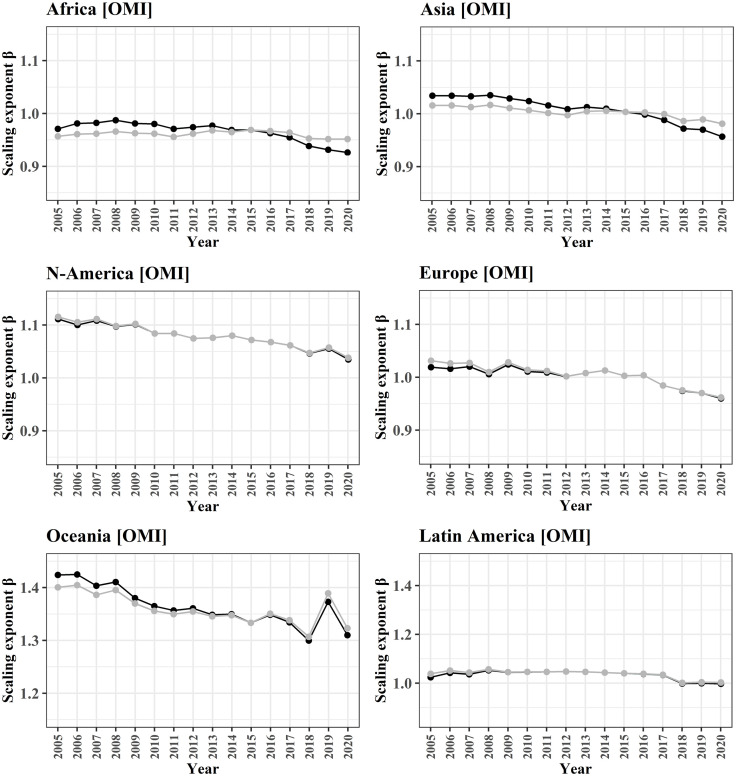
Effects of population count development over time on the scaling exponent. Data shows the scaling exponents between 2005 and 2020 for all cities with a minimum population size of 50,000 inhabitants. Scaling exponents were calculated between satellite-derived NO_2_ levels for each year and yearly population counts (black line). To demonstrate the relevance of temporally matching population counts for each year, the grey lines report scaling exponents with only the population counts from 2015, as reported in the UCDB. Largest effects of varying scaling relation due to varying population counts are observed for Africa and Asia.

In the previous analyses above we addressed various characteristics of NO_2_ data and spatial entities, while the population size of the cities was used as a stable variable as reported for the year 2015. In some regions, however, we observe stagnating NO_2_ levels in combination with a stark increase of population counts (Africa), or decreasing NO_2_ levels in combination with increasing population counts (Asia). To account for temporal effects of changing population sizes on the scaling relations, we repeated the scaling analysis for the time period 2005−2020 with linearly extrapolated population data until 2020 based on population trends from 2000−2015 as reported in the UCDB data set. Results of this analysis are depicted in (Fig 11) and show partial effects on the scaling estimates *β* which are induced by temporally mismatched population data with NO_2_. The maximum observed differences in scaling estimates (Δ_max_) are + 0.014/-0.025 for Africa and +0.020/-0.024 for Asia. Even though the effects are little, we observe that the use of static population data may lead to biased scaling exponents in fast growing urban areas.

## 5. Summary and discussion

This study is motivated by a recently observed rising scientific interest in the evaluation whether larger cities experience better or worse ambient air quality (see Section 1). The concept of urban scaling laws provides an appropriate method for the systematic analysis of urban characteristics and their behavior with increasing city size. The main body of scientific literature in scaling of urban air quality, however, is ambiguous in their main findings since the studies report varying, even contradicting results. Some reasons for this can be related to varying data sources and different spatial concepts for the analysis. Both, an objective data source for ambient urban air quality and a globally comparable, harmonized spatial definition of cities are important for the generation of reproducible and meaningful results.

In this paper, we add to the current body of literature by tackling some of the most critical fallacies in studying global scaling of urban air quality. Our analysis fills gaps in current literature by *using global satellite-based observations of nitrogen dioxide at unprecedented spatial and temporal resolution as a short-lived tracer for urban air quality* in combination with a *globally harmonized definition of urban boundaries of more than 10,000 cities in the world using fine-grained population data generated from satellite images.* Both data sets ensure a maximum of objectivity and unlimited spatial transferability, which are key prerequisites to global comparability of urban air quality. Satellite-based observations of NO_2_ represent the actual number of nitrogen dioxide molecules at a certain point in time for a certain area above the Earth’s surface. Through repeated observations over years and decades, the data allow for the detection of long-term trends and short-term variations [48,61]. Observations of NO_2_ from satellites are typically lower than in-situ or ground-based measurements [45], but these effects are identical around the globe, thus making satellites a relatively objective data source without regional or political bias. Despite the shortcomings of using tropospheric NO_2_ column densities for ambient NO_2_, we prefer observations over model-inferred surface-levels from satellite-based observations [47]. In particular the urban areas of smaller cities are yet not well represented in global models and this would therefore result in a bias towards larger cities which might jeopardize the scaling study.

We specifically analyze and compare available data on nitrogen dioxide from various satellite missions and emission inventory data at global scale. In this context, several important data characteristics are analyzed to define the best study set-up, such as data source and data granularity (Section 3.1), and temporal effects (Section 3.3). In this context, the results of our experiments indicate that NO_2_ observations from the Ozone Monitoring Instrument (OMI) at a spatial sampling of 0.125° × 0.125° represent a reliable, stable data source for global analyses of urban air quality since 2024. It provides the longest consistent time series at an acceptable spatial resolution for global analyses.

Since meteorological effects impact air quality and may interfere yearly aggregates of NO_2_ levels, a temporal aggregation of data over multiple years reduces effects of data outliers. At the same time, global urban population increases while in many countries national policies for the reduction of NO_2_ emissions lead to a long-term decrease of air pollutants. Therefore, a careful selection of the used time periods for population data and for the aggregates of NO_2_ estimates is crucial as we can see by the example of scaling estimates for Asia which are inverted between the year 2005 and 2015 (Fig 11).

Due to a significant level of background NO_2_ which is measured from satellites, the spatial definition of urban boundaries impacts the scaling relation between population and NO_2_ in the form that larger urban areas incorporate higher sums of NO_2_ even if some areas might not be built-up and thus no human-related combustion processes take place. Similar effects have been observed for some urban properties [34,35] and in particular for CO_2_ emissions [24]. We therefore recommend a narrow-tailored delimitation of urban areas based on population density [36,37] to only include populated areas and as little background NO_2_ as possible.

Based on the detailed analysis of input data, we defined the best fitting, unbiased experimental set-up for a global analysis of urban air quality scaling. The entire data set using >10,000 cities with more than 50,000 inhabitants clearly reveals a strong global linear scaling behavior (*β = 0.999*). This result indicates that at a global level no positive (economy of scale, sub-linear) or negative effects (increased returns to scale, super-linear) of a city’s population size on the NO_2_ air pollution can be observed. Thus, globally, NO_2_ levels for cities increase directly proportional with the number of inhabitants of a city which means that larger cities are neither greener nor more polluted than smaller cities. Based on this single analysis, one could assume a universal, globally valid linear scaling behavior. Even when we analyze the scaling relationships for the individual continents, no significant deviations from linearity can be observed.

On a spatially more detailed level, however, we observe strongly varying scaling behaviors around the world. When the same analysis is performed for smaller geographical entities such as sub-continental regions, or countries, we identify more pronounced intra-continental variations of scaling exponents between sub-linear and super-linear scaling for each continent. The largest range of variations of observed scaling exponents are reported for regions in Africa (*0.788–1.118*).

At the smallest scale of individual countries, we observe also highest variations of scaling exponents (*0.693–1.634*) together with better, because locally fitted, coefficients of determinations. Moreover, the analysis indicates that the scaling exponent *β* is non-universal. Similar observations have been previously reported also for CO_2_ [25]. Some indications which may have an impact on the NO_2_ efficiency of a city can be found in socio-economic grounds, such as the income level which follows an Environmental Kuznets Curve.

While at the national level for the U.S. some related studies report larger cities to perform less favorably in terms of CO_2_ efficiency [24], it is due to our findings not appropriate to draw conclusions to the global scale based on a national sub-sample. For the U.S., our observations show also a super-linear scaling behavior, however, at the global level, we cannot find evidence in the data that NO_2_ levels increase or decrease with rising population sizes of cities. Our findings underscore the need for caution when generalizing urban scaling results. City size alone does not universally predict NO₂ efficiency. The relation between city size and NO_2_ levels seems to be more complex and non-universal. Therefore, we first assume a stronger role of national or regional policies on emissions around the world like environmental taxes [78]. Second, the urban configuration type exhibits a significant influence on the relation between NO_2_ air pollution and population density as has recently been substantiated for 919 cities in Europe by [11]. Third, the effects of commuting also alter the NO_2_ burden regarding the degree of urbanization [6]. Finally, especially for some developing countries, we found better coefficients of determination between city area and NO_2_ levels than with population numbers. While the used data set with globally gridded population at 1 km spacing belongs yet to the currently most fine-grained and detailed data sets, the quality of the data source is impacted by out-dated or incorrect census data or by limitations of the underlying settlement mask from satellite images. Image classification accuracies may vary significantly around the globe for the task of impervious area mapping and are specifically problematic in Africa [79]. In the near future, finer-grained and more reliable global settlement masks and population data will shed some light on this particular issue, e.g., WorldPop [80] or the 3D World Settlement Footprint [81]. These data could be used to compare effects on the mapping of urban centers and possible impacts on the scaling analyses.

While some individual aspects of this study were reported in previous studies, this work represents a comprehensive and systematic evaluation of global scaling of urban air quality because of the unique and unprecedented data sets which have been used and the focus on the global application for more than 10,000 cities.

## 6. Conclusions

With more and more people migrating into urban areas [8], the future of our planet will be decided in cities. In the light of global change and increased environmental awareness, the debate whether larger cities are environmentally greener than smaller cities has intensified. From a theoretical perspective but also from practical examples, we know that we can benefit from high concentrations of people in cities. This is because some resources can be used more efficiently which in turn can help to be less harmful to the environment [82]. The concept of urban scaling has shown that for many urban attributes, urban systems can benefit from economies of scale (e.g., number of gas stations, road lengths) or increased returns to scale (e.g., wages, number of patents) [16]. Application of this concept has attracted increased interest in related scientific disciplines to a wide spectrum of urban attributes. The variety of results, however, substantiates that the concept is difficult to apply to any urban feature. Therefore, a better understanding of the relation of NO_2_-levels and urban areas is needed to help mitigating ambient air pollution as a major threat to human health worldwide, especially as there exists no generally agreed set of urban and socioeconomic properties which may impact urban emissions on a global scale. Especially the role of density, urban form and urban structure on urban emissions and pollution levels open up new paths for future research [23,25,40,69,83]. Another relevant aspect for directing future work relates to the role of income or economic development status [19,24,25,69] and national policy implications on urban emissions as national or super-national authorities have the greatest power to change the behavior of urban residents. This can be seen by the effects of newly introduced laws on emissions in, e.g., Nordic countries or the evolution of the trends for NO_2_-levels in Chinese cities, where despite massive urban growth, NO_2_-levels at the country level were observed to be decreasing after environmental regulations were applied, or the European Green Deal which aims at severely cutting down emissions by 2050.

This study highlights the potential of satellite-based observations of tropospheric NO₂ for analyzing scaling effects of urban air quality. Despite the original design of early instruments such as GOME for stratospheric ozone monitoring, they have demonstrated sufficient sensitivity to detect tropospheric NO₂ signals. One of the primary strengths of these observations lies in their capability to provide consistent, global coverage that facilitates cross-regional and temporal comparisons.

However, several limitations affect the robustness and resolution of satellite-derived NO₂ data. Key constraints include relatively coarse spatial resolution compared to urban scales, limited temporal resolution (typically one daytime overpass per day) and dependency on solar backscatter (thus constrained by daylight and cloud cover). In particular, the coarse resolution of GOME-2A exhibits a tendency to mix urban NO₂ signals with surrounding rural areas, diluting the urban signal crucial for accurate scaling analysis. However, the results obtained are consistent with sensor of the latest generation like TROPOMI with the highest spatial resolution achieved so far. Moreover, the satellite instruments retrieve vertical column densities, not direct surface-level concentrations, which adds to the complexity of interpretation.

Regarding uncertainties, tropospheric NO₂ column retrievals are affected by various error sources, including cloud fraction, surface albedo, a priori NO₂ profiles, and atmospheric parameters used in the retrieval process. While uncertainties in individual observations can be high—up to 80% under polluted conditions—the use of temporal averaging significantly reduces random errors. Nevertheless, systematic biases persist, particularly under high-pollution conditions, where satellite products tend to underestimate ground-level NO₂ observations.

Despite these challenges, the relatively short atmospheric lifetime of tropospheric NO₂ ensures that its satellite-observed column densities predominantly reflect local emission sources. Therefore, satellite-based NO₂ observations are particularly well-suited for analyzing air pollution hotspots like cities and the associated urban scaling effects.

## Supporting information

S1 Table (docx)
