# Supplementary material for: Global scaling of urban air quality
Source: PLoS One. 2025 Oct 13;20(10):e0333902. doi: 10.1371/journal.pone.0333902 (PMC12517508; doi:10.1371/journal.pone.0333902)
Supplement: S1 Table — (docx) [file pone.0333902.s001.docx]

**Appendix** **Scaling results for each country**

**S1 Table.** **Coefficients of scaling results by countries of the world**. Listed are only countries with a minimum of 2 cities with more than 50,000 inhabitants. Coefficient of determination (R²), and confidence interval (CI) is reported only for countries with more than 3 cities. Abbreviations for continents: Asia (AS), Africa (AF), Europe (EU), Latin America (LA), Northern America (NA), Oceania (OC).

| ISO 3166 | Name | Cont. | β | R² | CI | Cities |  | ISO 3166 | Name | Cont. | β | R² | CI | Cities |
| --- | --- | --- | --- | --- | --- | --- | --- | --- | --- | --- | --- | --- | --- | --- |
| AFG | Afghanistan | AS | 0.978 | 0.689 | 0.246 | 29 |  | KOR | South Korea | AS | 0.785 | 0.812 | 0.122 | 39 |
| AGO | Angola | AF | 0.923 | 0.735 | 0.163 | 47 |  | KWT | Kuwait | AS | 0.883 | 0.998 | 0.050 | 4 |
| ALB | Albania | EU | 0.933 | 0.868 | 0.320 | 6 |  | **LAO** | Laos | AS | 0.919 | 0.990 | 0.109 | 4 |
| ARE | Un. Arab Em. | AS | 1.084 | 0.839 | 0.382 | 7 |  | **LBN** | Lebanon | AS | 0.852 | 0.861 | 0.275 | 7 |
| ARG | Argentina | LA | 1.061 | 0.734 | 0.152 | 71 |  | **LBR** | Liberia | AF | 0.893 | 0.917 | 0.265 | 5 |
| ARM | Armenia | AS | 1.154 | 0.998 | 0.076 | 3 |  | **LBY** | Libya | AF | 1.254 | 0.732 | 0.400 | 15 |
| AUS | Australia | OC | 1.135 | 0.888 | 0.158 | 27 |  | **LKA** | Sri Lanka | AS | 1.165 | 0.887 | 0.190 | 20 |
| AUT | Austria | EU | 1.020 | 0.902 | 0.298 | 6 |  | **LTU** | Lithuania | EU | 0.942 | 0.945 | 0.202 | 6 |
| AZE | Azerbaijan | AS | 0.950 | 0.940 | 0.128 | 15 |  | **LVA** | Latvia | EU | 1.031 | 0.959 | 0.300 | 3 |
| BDI | Burundi | AF | 0.905 | 0.701 | 0.301 | 16 |  | **MAR** | Morocco | AF | 1.031 | 0.844 | 0.114 | 61 |
| BEL | Belgium | EU | 0.837 | 0.897 | 0.170 | 12 |  | **MDA** | Moldova | EU | 0.907 | 0.953 | 0.201 | 5 |
| BEN | Benin | AF | 1.133 | 0.845 | 0.216 | 21 |  | **MDG** | Madagascar | AF | 0.989 | 0.856 | 0.283 | 9 |
| BFA | Burkina Faso | AF | 1.131 | 0.810 | 0.196 | 32 |  | **MEX** | Mexico | LA | 0.993 | 0.763 | 0.086 | 168 |
| BGD | Bangladesh | AS | 1.011 | 0.665 | 0.156 | 85 |  | **MKD** | Macedonia | EU | 1.080 | 0.958 | 0.183 | 7 |
| BGR | Bulgaria | EU | 0.862 | 0.940 | 0.177 | 7 |  | **MLI** | Mali | AF | 0.985 | 0.947 | 0.121 | 16 |
| BIH | Bosnia & Her. | EU | 0.702 | 0.831 | 0.309 | 5 |  | **MMR** | Myanmar | AS | 0.933 | 0.622 | 0.149 | 99 |
| BLR | Belarus | EU | 0.816 | 0.890 | 0.159 | 14 |  | **MOZ** | Mozambique | AF | 1.020 | 0.634 | 0.225 | 48 |
| BOL | Bolivia | LA | 1.118 | 0.736 | 0.380 | 13 |  | **MRT** | Mauritania | AF | 1.082 | 0.811 | 0.508 | 5 |
| BRA | Brazil | LA | 0.947 | 0.678 | 0.070 | 349 |  | **MWI** | Malawi | AF | 0.953 | 0.929 | 0.198 | 8 |
| BWA | Botswa- | AF | 1.634 | 0.764 | 0.723 | 7 |  | **MYS** | Malaysia | AS | 0.984 | 0.528 | 0.311 | 36 |
| CAF | Cen. Afr. Rep. | AF | 0.834 | 0.925 | 0.211 | 6 |  | **NAM** | Namibia | AF | 1.404 | - | - | 2 |
| CAN | Canada | NA | 1.041 | 0.822 | 0.141 | 48 |  | **NER** | Niger | AF | 0.788 | 0.639 | 0.230 | 27 |
| CHE | Switzerland | EU | 0.929 | 0.900 | 0.159 | 16 |  | **NGA** | Nigeria | AF | 1.129 | 0.692 | 0.076 | 392 |
| CHL | Chile | LA | 1.074 | 0.597 | 0.309 | 33 |  | **NIC** | Nicaragua | LA | 0.997 | 0.664 | 0.386 | 14 |
| CHN | China | AS | 1.077 | 0.524 | 0.048 | 1809 |  | **NLD** | Netherlands | EU | 0.965 | 0.870 | 0.124 | 37 |
| CIV | Côte d'Ivoire | AF | 0.993 | 0.879 | 0.126 | 35 |  | **NOR** | Norway | EU | 1.222 | 0.900 | 0.463 | 4 |
| CMR | Cameroon | AF | 0.755 | 0.666 | 0.160 | 45 |  | **NPL** | Nepal | AS | 0.952 | 0.887 | 0.204 | 12 |
| COD | D.R. Congo | AF | 0.744 | 0.474 | 0.139 | 127 |  | **NZL** | New Zealand | OC | 1.072 | 0.882 | 0.293 | 8 |
| COG | Rep Congo | AF | 1.034 | 0.674 | 0.467 | 10 |  | **OMN** | Oman | AS | 1.228 | 0.851 | 0.322 | 11 |
| COL | Colombia | LA | 0.946 | 0.693 | 0.134 | 89 |  | **PAK** | Pakistan | AS | 0.862 | 0.568 | 0.102 | 218 |
| COM | Comoros | AF | 1.998 | - | - | 2 |  | **PAN** | Panama | LA | 0.849 | 0.911 | 0.235 | 6 |
| CRI | Costa Rica | LA | 0.836 | 0.873 | 0.435 | 3 |  | **PER** | Peru | LA | 1.193 | 0.837 | 0.162 | 43 |
| CUB | Cuba | LA | 1.089 | 0.866 | 0.201 | 19 |  | **PHL** | Philippines | AS | 0.908 | 0.608 | 0.153 | 91 |
| CYP | Cyprus | AS | 0.799 | 0.844 | 0.465 | 3 |  | **POL** | Poland | EU | 1.047 | 0.846 | 0.133 | 46 |
| CZE | Czech Rep. | EU | 0.874 | 0.904 | 0.171 | 12 |  | **PRI** | Puerto Rico | LA | 0.693 | 0.671 | 0.615 | 3 |
| DEU | Germany | EU | 1.001 | 0.920 | 0.064 | 87 |  | **PRK** | North Korea | AS | 1.013 | 0.649 | 0.166 | 81 |
| DNK | Denmark | EU | 0.905 | 0.968 | 0.189 | 4 |  | **PRT** | Portugal | EU | 1.044 | 0.858 | 0.298 | 9 |
| DOM | Dom. Rep. | LA | 0.974 | 0.862 | 0.200 | 16 |  | **PRY** | Paraguay | LA | 1.215 | 0.960 | 0.188 | 8 |
| DZA | Algeria | AF | 1.123 | 0.552 | 0.208 | 95 |  | **PSE** | Palestine | AS | 0.751 | 0.972 | 0.104 | 7 |
| ECU | Ecuador | LA | 1.017 | 0.719 | 0.235 | 31 |  | **QAT** | Qatar | AS | 1.089 | 0.971 | 0.265 | 3 |
| EGY | Egypt | AF | 0.863 | 0.649 | 0.093 | 185 |  | **ROU** | Romania | EU | 0.855 | 0.769 | 0.176 | 29 |
| ERI | Eritrea | AF | 0.936 | 0.439 | 0.785 | 7 |  | **RUS** | Russia | EU | 0.998 | 0.721 | 0.087 | 204 |
| ESH | W. Sahara | AF | 0.784 | - | - | 2 |  | **RWA** | Rwanda | AF | 1.196 | 0.817 | 0.454 | 7 |
| ESP | Spain | EU | 0.976 | 0.847 | 0.098 | 72 |  | **SAU** | Saudi Arabia | AS | 1.154 | 0.752 | 0.193 | 48 |
| EST | Estonia | EU | 1.133 | - | - | 2 |  | **SDN** | Sudan | AF | 0.864 | 0.526 | 0.187 | 77 |
| ETH | Ethiopia | AF | 0.765 | 0.249 | 0.172 | 237 |  | **SEN** | Senegal | AF | 1.003 | 0.823 | 0.175 | 29 |
| FIN | Finland | EU | 1.344 | 0.983 | 0.158 | 6 |  | **SLE** | Sierra Leone | AF | 0.786 | 0.801 | 0.273 | 9 |
| FRA | France | LA | 1.009 | 0.656 | 0.168 | 76 |  | **SLV** | El Salvador | LA | 0.996 | 0.927 | 0.197 | 9 |
| GAB | Gabon | AF | 1.307 | 0.976 | 0.288 | 3 |  | **SOM** | Somalia | AF | 0.864 | 0.612 | 0.303 | 21 |
| GBR | Un. Kingd. | EU | 0.981 | 0.829 | 0.076 | 138 |  | **SRB** | Serbia | EU | 0.973 | 0.750 | 0.320 | 13 |
| GEO | Georgia | AS | 0.744 | 0.809 | 0.352 | 5 |  | **SSD** | South Sudan | AF | 0.797 | 0.397 | 0.444 | 19 |
| GHA | Ghana | AF | 1.021 | 0.718 | 0.180 | 51 |  | **SVK** | Slovakia | EU | 1.015 | 0.875 | 0.338 | 6 |
| GIN | Guinea | AF | 0.825 | 0.843 | 0.177 | 17 |  | **SVN** | Slovenia | EU | 1.237 | - | - | 2 |
| GMB | Gambia | AF | 1.114 | 0.813 | 0.594 | 4 |  | **SWE** | Sweden | EU | 1.011 | 0.904 | 0.198 | 12 |
| GNB | Guinea-Bissau | AF | 0.907 | 0.887 | 0.443 | 3 |  | **SWZ** | Swaziland | AF | 0.641 | - | - | 2 |
| GNQ | Equ. Guinea | AF | 0.413 | - | - | 2 |  | **SYR** | Syria | AS | 0.932 | 0.832 | 0.174 | 24 |
| GRC | Greece | EU | 1.003 | 0.958 | 0.139 | 10 |  | **TCD** | Chad | AF | 0.898 | 0.489 | 0.335 | 30 |
| GTM | Guatemala | LA | 0.917 | 0.458 | 0.318 | 39 |  | **TGO** | Togo | AF | 0.991 | 0.725 | 0.322 | 15 |
| GUY | Guyana | LA | 0.268 | - | - | 2 |  | **THA** | Thailand | AS | 1.085 | 0.663 | 0.237 | 43 |
| HND | Honduras | LA | 1.025 | 0.754 | 0.334 | 13 |  | **TJK** | Tajikistan | AS | 0.958 | 0.469 | 0.524 | 15 |
| HRV | Croatia | EU | 1.046 | 0.968 | 0.169 | 6 |  | **TKM** | Turkmenist. | AS | 0.960 | 0.662 | 0.445 | 10 |
| HTI | Haiti | LA | 1.113 | 0.799 | 0.248 | 21 |  | **TTO** | Trin. & Tob. | LA | 0.900 | 0.912 | 0.318 | 4 |
| HUN | Hungary | EU | 0.908 | 0.876 | 0.214 | 11 |  | **TUN** | Tunisia | AF | 1.325 | 0.861 | 0.212 | 26 |
| IDN | Indonesia | AS | 1.102 | 0.522 | 0.119 | 318 |  | **TUR** | Turkey | AS | 1.032 | 0.765 | 0.100 | 131 |
| IND | India | AS | 0.931 | 0.582 | 0.036 | 1923 |  | **TWN** | Taiwan | AS | 0.970 | 0.881 | 0.159 | 21 |
| IRL | Ireland | EU | 0.973 | 0.933 | 0.259 | 5 |  | **TZA** | Tanzania | AF | 0.969 | 0.849 | 0.129 | 41 |
| IRN | Iran | AS | 1.130 | 0.633 | 0.130 | 176 |  | **UGA** | Uganda | AF | 1.060 | 0.831 | 0.203 | 23 |
| IRQ | Iraq | AS | 0.935 | 0.701 | 0.147 | 70 |  | **UKR** | Ukraine | EU | 0.900 | 0.806 | 0.101 | 78 |
| ISR | Israel | AS | 0.896 | 0.855 | 0.258 | 9 |  | **URY** | Uruguay | LA | 0.930 | 0.897 | 0.279 | 6 |
| ITA | Italy | EU | 1.116 | 0.629 | 0.180 | 91 |  | **USA** | United States | NA | 1.055 | 0.876 | 0.044 | 324 |
| JAM | Jamaica | LA | 1.011 | 0.741 | 0.653 | 4 |  | **UZB** | Uzbekistan | AS | 0.892 | 0.673 | 0.167 | 56 |
| JOR | Jordan | AS | 1.007 | 0.907 | 0.227 | 9 |  | **VEN** | Venezuela | LA | 0.994 | 0.747 | 0.136 | 73 |
| JPN | Japan | AS | 0.941 | 0.801 | 0.090 | 109 |  | **VNM** | Vietnam | AS | 1.088 | 0.537 | 0.176 | 132 |
| KAZ | Kazakhstan | AS | 1.032 | 0.705 | 0.260 | 27 |  | **XKO** | Kosovo | EU | 1.284 | 0.909 | 0.328 | 7 |
| KEN | Kenya | AF | 0.833 | 0.451 | 0.283 | 42 |  | **YEM** | Yemen | AS | 0.988 | 0.801 | 0.189 | 28 |
| KGZ | Kyrgyzstan | AS | 1.001 | 0.677 | 0.475 | 9 |  | **ZAF** | South Africa | AF | 1.107 | 0.675 | 0.176 | 77 |
| KHM | Cambodia | AS | 0.903 | 0.929 | 0.188 | 8 |  | **ZMB** | Zambia | AF | 1.189 | 0.738 | 0.232 | 38 |
|  |  |  |  |  |  |  |  | **ZWE** | Zimbabwe | AF | 1.014 | 0.661 | 0.329 | 20 |
